# Measuring the missing: Knowledge, risk perceptions and self-protection practices of COVID-19 among the Asian population in New Zealand: An online survey

**DOI:** 10.1007/s10389-023-01926-0

**Published:** 2023-05-18

**Authors:** Liangni Sally Liu, Xiaoyun Jia, Andrew Zhu, Guanyu Jason Ran, David Johnston, Richard Siegert, Yuan Gong, Nigel French, Jun Lu

**Affiliations:** 1 School of Humanities, Media and Creative Communication, Massey University, Auckland, New Zealand; 2 Institute of Governance & School of Politics and Public Administration, Shandong University, Qingdao, China; 3 Trace Research Ltd, Auckland, New Zealand; 4 School of Applied Sciences, Edinburgh Napier University, Edinburgh, UK; 5 Joint Centre for Disaster Research, Massey University, Wellington, New Zealand; 6 Department of Psychology & Neuroscience, School of Clinical Sciences, Auckland University of Technology, Auckland, New Zealand; 7 Infectious Diseases Research Centre, Hopkirk Research Institute, Massey University, Palmerston North, New Zealand; 8 Auckland Bioengineering Institute, The University of Auckland, Auckland, New Zealand

**Keywords:** Risk perceptions, self-protection practices, COVID-19 pandemic, Asians, New Zealand

## Abstract

**Aim:**

Asians are the second largest and fastest growing non-European population in New Zealand but are under-researched in terms of their COVID-19 pandemic response. The paper aims to illustrates Asians’ risk perceptions and knowledge of COVID-19, and self-protection practices to avoid infection and prevent community transmission.

**Subject and methods:**

An online survey was used to collect data and received 402 valid responses. Data analyses included: 1) a descriptive analysis by using *Chi*-square tests and a Kruskal-Wallis rank sum tests to explore associations between responses and the four demographic variables (i.e. age, gender, country of origin/ethnicity, and region); and 2) a correlation analysis between different survey objectives.

**Results:**

The descriptive analysis of the survey found that while ethnicity (within the Asian category) was the most influential variable that resulted in varying responses to many questions, gender and age were other two important variables in influencing the answering patterns. The correlation analysis found a positive correlation between the perceived ‘dangerousness’ of COVID-19 and respondents’ overall compliance behaviour to New Zealand authorities’ recommendations to prevent spread of COVID-19.

**Conclusion:**

The majority of the respondents provided correct answers to the questions about the vulnerable populations, symptoms, asymptomatic transmission and potential sequelae of COVID-19; however, their understanding of the availability of a cure for, and the incubation period of COVID-19 was not consistent with the official information. The research also found that the higher perceived dangerousness of COVID-19, the better compliance to self-protection practices among the surveyed population.

## Introduction

In the first 18 months of the COVID-19 pandemic, given the fact that COVID-19 was a relatively new respiratory disease and the absence of effective treatments to it (Betsch 2020), the containment of the COVID-19 pandemic relied on population engagement in nonpharmaceutical interventions (Jefferies et al. 2020; Sebhatu et al. 2020). At the individual level, these interventions included requiring people to engage in prosocial action, including behaviours that may inconvenience individuals, but protect the collective, such as movement restriction, social distancing, hygiene practices, and contact tracing (Orom et al. 2021). In practice, how well these preventive methods and behaviours can be pursued is influenced by people's knowledge, risk perceptions and understanding of the disease (Bowman et al. 2021; Wise et al. 2020; Zhang et al. 2020), as well as by people’s anxiety levels, historical exposure to contagious diseases, social and cultural worldviews of individualism, and government responses and trust (Dryhurst et al. 2020; Nino et al. 2021). These cognitive outcomes of COVID-19, however, may vary by sociodemographic factors, such as gender, age, income, education, ability to access to COVID-19-related information and health literacy, and race and ethnicity (Geldsetzer 2020; Reiter and Katz 2021). So far limited research has been conducted on the role that race/ethnicity plays in determining people’s cognitive outcomes of COVID-19 and self-protection practices in the early stage of this pandemic.

Limited research has been undertaken on the relationship/association between race and ethnicity and people’s knowledge, attitudes and beliefs about COVID-19, and this has mostly taken place in the United States (US), United Kingdom (UK) and other Western European countries (Jamieson et al. 2021; Jones et al. 2020; Nino et al. 2021; Reiter and Katz 2021; Soine et al. 2021). Research conducted in US focused on black and Hispanic/Latinx. Reiter and Katz (2021)’s research reveals that certain ethnic minority groups, tended to provide less correct answers to questions about the causes and symptoms of COVID-19 compared with non-Latinx white. Jamieson et al. (2021) found that individuals who identified as Hispanic/Latinx and first-generation immigrants reported significantly greater health risks of becoming infected and dying from the virus if they do contract it. However, few studies have evaluated the health literacy levels of the Asian population.

Of the limited studies that consider Asian populations, Asians have been often placed into comparison with other ethnic populations, such as black and Hispanic/Latinx. In general, Asians seemed to have better knowledge of COVID-19 and intend to perceive the virus to be a major threat to their health (Jones et al. 2020; Nino et al. 2021; Niu et al. 2020). One study conducted in Germany to investigate ethnic groups’ risk perceptions (Soine et al. 2021) found that both health and financial risk perceptions were more prevalent in populations with a former Yugoslavian and Turkish background compared to German majority; however, Asians only presented their concerns of health risk, but not financial risk. Only one paper identified in our literature review divided the Asian population into sub-groups (including Chinese, Indians, Malays), and found that Indians and Malays in Singapore are much more likely to think they will avoid getting sick from COVID-19 compared to Chinese (Teo et al. 2021).

Many studies found a strong correlation between people’s perceptions of COVID-19 and their adoption of preventive behaviours. For example, one research found that during the early phase of the COVID-19 outbreak, Hong Kong reported higher level of adoption of social distancing and mask wearing; however, there was no correlation between the respondents’ perceived severity and adoption of social distancing and wearing a mask. In contrast, in the UK, those who perceived severity as ‘high’ were more likely to adopt social distancing and contact avoidance (Bowman et al. 2021). Research conducted in New Zealand on people’s self-reported belief of face covering during the country’s Alert Level 4 lockdown in 2020 found that those who viewed face covering as effective in preventing them from contracting COVID-19 were substantially more likely to report having worn a face mask than those who view face covering as ‘not at all’ effective. Among all respondents, Asians had reported the highest percentage of mask wearing, higher than New Zealand Europeans, Māori and Pacific people, and people of other race/ethnicity (Gray et al. 2020).

The literature discussed above illustrates 1) how broader racialised social structures shape socioeconomic and health disparities and influence the pandemic knowledge and risk perceptions as well as self-protection practices, and 2) how people’s perceptions of COVID-19 interact with their self-protection practices. The reality that COVID-19 has disproportionately affected disadvantaged ethnic communities is well acknowledged (Arena et al. 2020; Lopez et al. 2021; Sze et al. 2020). Existing research also finds that structural racism has enabled dominant ethnic groups to accumulate and access a set of flexible resources; however, historically marginalised racial and ethnic groups unfortunately tend to have limited access to medical information and receive poorer quality care (Phelan and Link 2015; Williams et al. 2019). Moreover, the lack of economic and educational opportunity has resulted in some ethnic minorities being overrepresented in high-risk jobs with higher chances of being exposed to infection (Rogers et al. 2020; Selden and Berdahl 2020).

In New Zealand, racial inequality in health has been well researched among Māori and Pacific populations. These two ethnic communities have been historically disadvantaged in public health (Paradies et al. 2015; Talamaivao et al. 2020) and negatively impacted by pandemics/epidemics (Steyn et al. 2021, Wilson et al. 2012). The major reasons for this higher proportion of infection and hospitalisation included the lack of high-quality suitable housing, poorer access to health care, and pre-existing medical conditions.

However, Asians, as the second largest and fastest growing non-European population in New Zealand, are relatively under-researched in terms of their COVID-19 pandemic outcomes. Among this population, 77% of them were born overseas, which means that the majority of the New Zealand Asian population are immigrants (Statistic New Zealand 2018). As for the COVID-19 pandemic, even though anecdotal evidence indicates that Asians’ previous experience with the SARS outbreak in 2003 has led them to carry heightened precautions in response to the current pandemic (Jones et al. 2020; Liu and Ran 2020); unfortunately, there has been no study which particularly focuses on this ethnic population in New Zealand and breaks this group down to different sub-groups to understand how the pandemic impacts on them differently. In this research, an online survey was conducted in New Zealand’s Asian population. It is the first study to investigate Asians’ risk perceptions and knowledge of COVID-19, and self-protection practices to avoid infection and prevent community transmission of COVID-19 in New Zealand. Asians in this research are defined as those adult Asian residents in New Zealand, including those who were born in Asian countries and born in New Zealand. They have already obtained New Zealand residency, permanent residency, or citizenship. Asian countries are defined by using the New Zealand Census standard. Those Western and Central Asian countries are not defined as Asian countries by the Census.

## Methods

### Sampling

An online survey was used to collect data. The research was approved by the Massey University’s Human Ethics Committees and judged to be low risk (Ethics Notification Number 4000022629). Informed consent for each participant has been obtained in the beginning webpage of the online survey. To be an eligible respondent, one must be over 18 years old and self-identified with a cultural origin from the Asian countries as defined by the New Zealand Census standard. People who cannot meet the above eligibility criteria are excluded from the survey. A random sample of the Asian adult population, stratified by age, ethnicity, gender and location, according to the 2018 New Zealand Census, was used to ensure representativeness of all Asian ethnic groups in the country. The stratified sampling technique meant that when the quota has met, the data collection for specified groups was stopped. This was the most effective way to meet all sampling criteria in a timely manner.

The researched subject, Asians, were identified by their countries of origin. Both overseas- and New Zealand-born Asians were included in the sampling. A power analysis was conducted before the data collection, it was determined that a sample size of 307 or more measurements is necessary to meet the desired statistical constraints. This sample size provided a 95% confidence level that the true value was within ±4% of the measured/surveyed value. Data were collected between the 5^th^ and 18^th^ of December 2020. The ethnic Chinese sample was collected through a contracted research company’s Chinese Immigrants Research Panel. All other Asian ethnic samples were collected from the contracted company’s partner online panel (by random email invitations of n=1,101, the total response rate was 36.5% when all quotas were filled). Only complete questionnaires without missing answers could be submitted for analysis. Each respondent was allowed to submit the questionnaire once only, according to the IP address recorded by the research panels.

### The sample

In total, there were 402 valid Asian respondents among which there were 31 New Zealand-born Asians, comprising 4.2% of the total sample (See Appendix Table 8 for a complete sample composition breakdown). Respondents were originally from more than 14 Asian countries/areas in which India and China were the first two largest source countries of the valid respondents. In detail, 135 (33.6%) respondents were with a cultural origin of India, while 126 (31.3%) respondents with a cultural origin of China. Respondents were residing across 17 regions in New Zealand. As the biggest city in New Zealand, the Auckland region hosts the largest respondent group, comprising 65.1% (namely, 262 respondents) of the final sample. This is followed by the Wellington region (52; 12.8%) and Canterbury region centred on Christchurch (28; 7.0%). The final sample is also gender balanced with 197 male, 203 female, and 2 ‘others’. As for the age distribution, the final sample is dominated by respondents who are in the age groups of 18-29 years (134; 33.4%) and 30-49 years (141; 35.1%), while respondents of 50-64 years comprise 23.4% (namely, 94 respondents) of the final sample. The smallest age group is those of 65+ years old, which comprises 8.1% (namely, 33 respondents) of the sample. Such socio-demographic characteristics of the respondents reflects on the true picture of the Asian population in New Zealand.

### Measurement

To measure Asian people’s knowledge about COVID-19, respondents were instructed to answer six COVID-19 related questions without checking the Internet or other sources. The questionnaire was developed based on the survey tool and guidance provided by WHO Regional Office for Europe (2020). All adopted scales were empirically validated by WHO Regional Office for Europe and cross-checked by an epidemiologist in New Zealand. These six questions aim to find out the perceived 1) COVID-19 vulnerable populations; 2) symptoms of COVID-19; 3) availability of a cure for COVID-19; 4) transmission of COVID-19; 5) incubation period of COVID-19, and 6) potential sequelae of COVID-19. Except for the open-ended Questions 5, ‘perceived incubation period of COVID-19’, which required respondents to enter answers manually; all were multiple response questions.

The risk perceptions are measured using two questions that ask about the 1) health impacts of COVID-19 on human (framed as ‘dangerousness of COVID-19’ in the survey questionnaire), and 2) susceptibility to infection with COVID-19, with responses based on a 10-point Likert scale ranging from -5 to +5.

The self-protective behaviours assessed in this research were drawn from both the survey tool and guidance provided by WHO Regional Office for Europe (2020) and the guidelines issued by the New Zealand government. Participants were asked to rate their overall compliance behaviour (i.e. ‘I follow the recommendations from authorities in New Zealand to prevent the spread of COVID-19’) on a 10-point Likert scale ranging from -5 (not at all) to +5 (very much so). Then participants were asked to choose all self-protective behaviours adopted to prevent infection and the spread of COVID-19.

### Data analysis

Data analysis was carried out using two approaches. Firstly, the characteristics of the surveyed respondents were described through frequency analysis and analyses of contingency tables. For non-ordered categorical variables, *Chi*-square tests were used to test for significant differences. For variables measured on continuous and ordered Likert scales, a Kruskal-Wallis rank sum test was used, followed by *Chi*-square tests and pairwise comparisons using a Wilcoxon rank sum test with continuity correction to test for independence among the respondents. The four key demographic variables of interest were: age, gender, country of origin (a proxy for ethnicity), and place of residency. The purpose of this part of analysis was to explore associations between responses and those four demographic variables. A *p* < 0.05 was considered statistically significant. To satisfy the requirements of conducting *Chi*-square tests, the answers with limited counts (such as ‘don’t know’, ‘other preventive measure’ and ‘using antibiotics’) were removed from the analysis. Two respondents who stated their gender as ‘others’ were also excluded from gender differences analyses. For the same reason, other than the three major ethnic groups (i.e. Chinese, Indians and Filipinos), other small Asian sub-groups (i.e. people from Hong Kong, Cambodia, Japan, Korea, Sri Lanka, Singapore, Malaysia, Thailand and ‘other Asian countries’) were re-grouped as ‘other Asians’, and people in the age groups of 50-64 years and of 65 years plus were re-grouped as the senior respondent group of ‘50 years and over’. We compared the analysis results with official information provided by relevant New Zealand authorities to assess whether the results are aligned well with the information published by the government.

The second part of the analysis investigates the hypothesised correlation between different survey objectives. A linear regression model was used to investigate the hypothesised correlation between the perceived health impacts of COVID-19 on respondents’ overall compliance behaviour to the New Zealand government’s recommendations. A *p* < 0.05 was considered statistically significant. The tested hypothesis is that a higher perceived dangerousness of COVID-19 will likely lead to a stronger compliance with health authorities.

Descriptive analyses were performed using SPSS 26 and the Kruskal-Wallis, Wilcoxon rank sum tests and correlation analyses were carried out in R and RStudio.

## Results from the descriptive analysis

### Knowledge about COVID-19

The survey found a relatively good knowledge of COVID-19 amongst the Asian respondents. This can be evidenced by their correct understanding of the vulnerable populations, symptoms, asymptomatic transmission and potential sequelae of COVID-19. The following will discuss these aspects one by one.

#### Perceived COVID-19 vulnerable populations

**Fig. 1:**
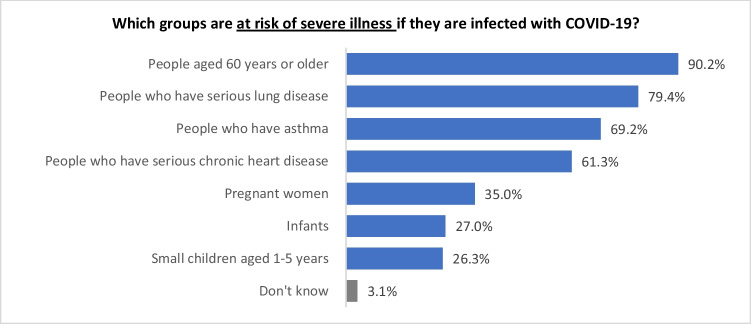
Perceived COVID-19 vulnerable populations

Most respondents (96.9%) provided answers to the knowledge assessment questions. Figure 1 shows that the Asian community believe that older people (aged 60 years or older) (90.2%) are most at risk of severe illness if infected with COVID-19, while those with existing health conditions are the next group deemed most at risk. In detail, 79.4% respondents thought that ‘people who have serious lung disease’, 69.2% respondents thought that ‘people who have asthma’, and 61.3% respondents thought that ‘people who have a serious chronic heart disease’ are at risk. In contrast, relatively few individuals believed that young children were at risk of severe illness, as evidenced that 27% respondents thought that infants are at risk and 26.3% respondents thought that children who are aged 1-5 years old are at risk. These numbers indicate the majority of respondents had a good knowledge of vulnerable populations.

To satisfy the expected cell size requirements for conducting *Chi*-square tests, age of responders was re-grouped into 18-29 years, 30-49 years and 50 years and over. Significant differences in perceptions of severity were evident for all four variables examined: ethnicity (χ^2^ = 37.6; df = 21; *p* = 0.014), gender (χ^2^ = 22.8; df = 7; *p* = 0.002), age (χ^2^ = 33.5; df = 14; *p* = 0.002) and region (χ^2^ = 45.2; df = 28; *p* = 0.021). Table 1 shows five variables that showed significant differences: 1) significantly more Chinese (89.1%) than Indians (75.7%) thought that ‘people who have serious lung disease’ are at risk of severe illness; 2) significantly more female (43.7% and 76.4%, respectively) than male (28.3% and 65.8%, respectively) chose that ‘pregnant women’ and ‘people who have asthma’ are groups at risk; 3) significantly more young people who are 18-29 years (37% and 35.3%, respectively) and 30-49 years (29.4% and 28.8%, respectively) thought that ‘infant’ and ‘small children aged 1-5 years’ are vulnerable groups compared with senior respondents who are 50 years old and over (15.8% and 16.1%, respectively); 4) significantly more respondents from Wellington region (90.6%) than those from South Island (59.7%) chose ‘people who have serious lung disease’ as vulnerable group; and 5) significantly more respondents from Wellington region (90.5%) than those from South Island (57.6%), Christchurch region (58.5%) and Auckland region (68.6%) thought ‘people who have asthma’ are vulnerable.

**Table 1:** Significant differences of respondents’ answers regarding their perceptions of COVID-19 vulnerable populations

Which groups are at risk of severe illness if they are infected with COVID-19?	Ethnicity				Gender		Age			Region				
	Chinese	Indians	Filipinos	Other Asians	Male	Female	18-29 years	30-49 years	50 years and over	AKL Region	WLG Region	CHC Region	Rest of NI	Rest of SI
	Column N %	Column N %	Column N %	Column N %	Column N %	Column N %	Column N %	Column N %	Column N %	Column N %	Column N %	Column N %	Column N %	Column N %
People aged 60 years or older	92.6%	92.2%	92.8%	93.1%	92.9%	92.2%	92.6%	92.9%	92.3%	93.0%	96.1%	89.8%	88.6%	91.3%
Pregnant women	33.6%	43.4%	33.3%	29.1%	28.3%	43.7%	38.7%	36.6%	32.2%	33.1%	41.0%	37.9%	46.2%	31.7%
Infants	29.1%	32.9%	16.6%	22.6%	24.9%	30.2%	37.0%	29.4%	15.8%	25.3%	32.1%	31.1%	35.8%	22.9%
Small children aged 1-5 years	27.1%	35.4%	14.0%	20.1%	27.3%	26.5%	35.3%	28.8%	16.1%	25.1%	29.2%	45.4%	28.8%	13.6%
People who have serious chronic heart disease	59.4%	64.4%	69.1%	63.6%	59.2%	67.3%	59.8%	65.8%	63.3%	61.8%	69.3%	64.9%	64.6%	53.6%
People who have serious lung disease	89.1%	75.7%	86.1%	76.2%	78.0%	85.2%	82.3%	83.2%	78.8%	81.2%	90.6%	79.7%	81.6%	59.7%
People who have asthma	78.0%	68.2%	67.7%	65.9%	65.8%	76.4%	70.5%	75.6%	66.6%	68.6%	90.5%	58.5%	75.5%	57.6%

#### Perceived symptoms of COVID-19

**Fig. 2:**
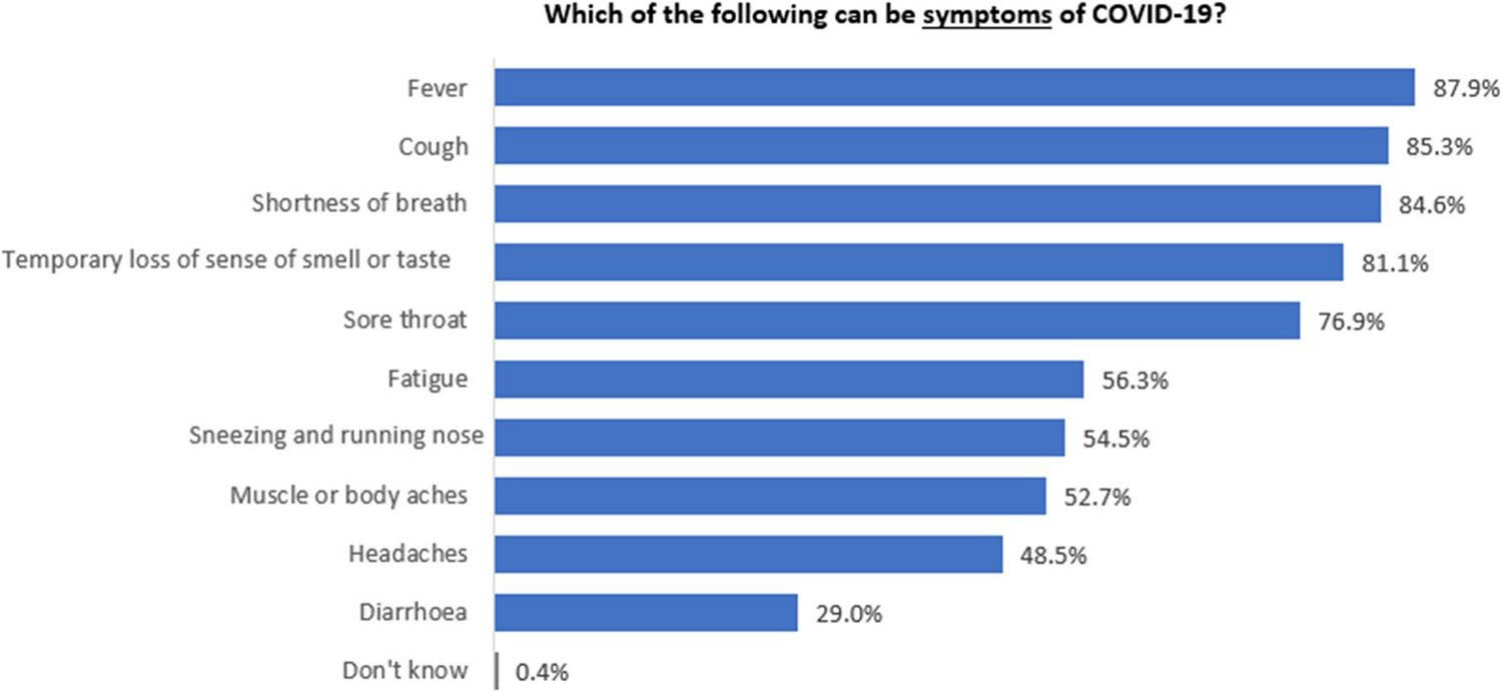
Perceived symptoms of COVID-19

Over 75% of the respondents identified five of the seven common symptoms of COVID-19 identified by the New Zealand Ministry of Health (MoH) (Ministry of Health of New Zealand 2022). They are fever, cough, shortness of breath, temporary loss of sense of smell or taste and sore throat (Fig. 2). Over 50% identified the remaining two common symptoms, fatigue, and sneezing and running nose, and one of the less common symptoms (i.e. muscle or body aches identified by the MoH). Two other symptoms were identified by fewer than 50% of respondents, namely headaches and diarrhoea, and these are also identified as uncommon symptoms by the MoH.

Perception of COVID-19 symptoms differed significantly between ethnic groups (χ^2^ = 74.1; df = 30; *p* <0.001 ) and gender (χ^2^ = 47.4; df = 10; *p*<0.001). Significantly more Chinese (91.7%) than ‘other Asians’ (79.2%) thought that ‘fever’ is COVID-19 symptom, significantly more Filipinos (97.8%) than Indians (78.6%) chose ‘cough’ as COVID-19 symptom, significantly more Chinese (65.2%) than ‘other Asians’ (47%) and Indians (43.5%) believed that ‘muscle or body aches’ is COVID-19 symptom, and significantly more Chinese (85.8%) than Indians (72.8%) thought that ‘temporary loss of sense of smell or taste’ is COVID-19 symptom (Table 2). As for gender, significantly more female (88.3%, 59.9%, 62.5%, 38.2% and 87%, respectively) than male (81.2%, 45.7%, 50.4%, 19.5% and 75.9%, respectively) thought ‘shortness of breath’, ‘muscle or body aches’, ‘fatigue’, ‘diarrhoea’ and ‘lost sense of smell’ are COVID-19 symptoms.

**Table 2:** Significant differences of respondents’ answers regarding their perceptions of COVID-19 symptoms

Which of the following can be the symptoms of COVID-19?	Ethnicity				Gender	
	Chinese	Indians	Filipinos	Other Asians	Male	Female
	Column N %	Column N %	Column N %	Column N %	Column N %	Column N %
Fever	91.7%	89.1%	91.2%	79.2%	86.8%	89.2%
Cough	88.1%	78.6%	97.8%	86.4%	82.5%	88.8%
Shortness of breath	85.9%	81.0%	95.6%	84.1%	81.2%	88.3%
Sore throat	75.1%	79.4%	86.1%	72.6%	75.2%	79.3%
Runny or stuff nose	53.2%	55.1%	63.3%	51.9%	53.8%	54.9%
Muscle or body aches	65.2%	43.5%	54.2%	47.0%	45.7%	59.9%
Headaches	51.2%	45.3%	60.8%	44.0%	46.1%	51.1%
Fatigue	58.0%	53.1%	70.3%	52.8%	50.4%	62.5%
Diarrhoea	35.7%	21.9%	40.3%	24.2%	19.5%	38.2%
Lost sense of smell	85.8%	72.8%	88.7%	84.0%	75.9%	87.0%

#### Perceived availability of vaccines and therapeutic agents for the prevention and treatment of COVID-19

**Fig. 3:**
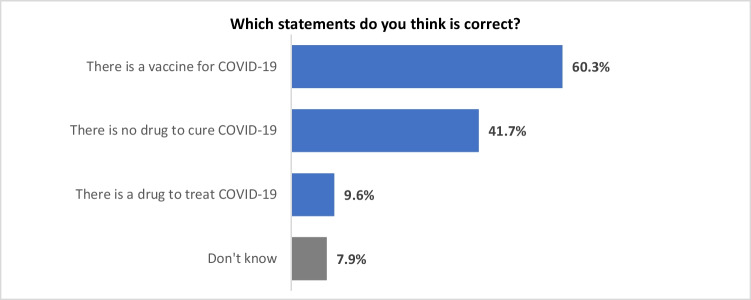
Perceived availability of cure for COVID-19

In terms of the prevention and treatment of COVID-19, 60% of respondents believed that ‘there is a vaccine for COVID-19’ and 42% believed that ‘there is no drug to cure COVID-19’. Only 9.6% respondents believed that ‘there is a drug to treat COVID-19’, and 7.9% respondents chose ‘do not know’ (Fig. 3). At the time the survey was carried out, COVID-19 vaccines were still under development and not available in New Zealand, and the evidence for effective treatments for COVID-19 was very weak. The first COVID-19 vaccine - Pfizer-BioNTech COVID-19 Vaccine was approved by the U.S. Food and Drug Administration (FDA) on 23 August 2021 (U.S. Food and Drug Administration 2021), and this was subsequently approved for use in New Zealand (New Zealand Medicines and Medical Devices Safety 2022). The optimistic reporting in the media leading towards the final approval of vaccine might have caused the respondents’ perception that there is a vaccine for COVID-19. For therapeutic agents, the FDA approved an antiviral drug called remdesivir (Veklury) to treat COVID-19 on 22 October 2020 (U.S. Food and Drug Administration 2020), just before this survey was conducted.

There was a significant difference between respondents’ perception of availability of therapeutic agents to treat or cure COVID-19 and ethnicity (χ^2^ = 69.9; df = 9; *p*<0.001). Table 3 indicates that significantly more Filipinos (16.3%) and ‘other Asians’ (14.8%) thought that ‘there is a drug to treat COVID-19’ than Chinese (4.1%). In addition, significantly more Chinese (71.3%) believed ‘there is no drug to cure COVID-19’ than other three ethnic groups (Indians - 31.9%, Filipinos - 21.2%, and ‘other Asian’ - 34%).

**Table 3:** Significant differences of respondents’ answers regarding their perceptions of availability of treatment or cure for COVID-19

What statements do you think is correct?	Ethnicity			
	Chinese	Indians	Filipinos	Other Asians
	Column N %	Column N %	Column N %	Column N %
There is a drug to treat COVID-19 - Which statements do you think is correct?	4.1%	12.4%	16.3%	14.8%
There is a vaccine for COVID-19 - Which statements do you think is correct?	61.5%	65.7%	71.9%	66.7%
There is no drug to cure COVID-19 - Which statements do you think is correct?	71.3%	31.9%	21.2%	34.0%

#### Perceived transmission of COVID-19

**Fig. 4:**
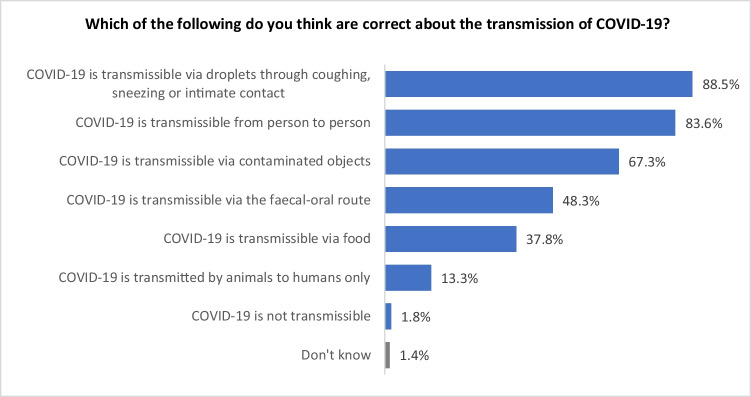
Ranking of perceived transmission of COVID-19

The overall ranking of perceived transmission of COVID-19 is presented in Fig. 4. The top three ways that the Asian respondents thought COVID-19 can be transmitted are via 1) droplets (i.e. through coughing, sneezing, or intimate contact); 2) person to person; and 3) contaminated objects. Among all respondents, 88.5% of them chose ‘via droplets through coughing, sneezing, or intimate contact’, 83.6% chose ‘from person to person’, and 67.3% chose ‘via contaminated objects’. Only a small proportion (1.8%) of the respondents believed COVID-19 cannot be transmitted. The survey findings demonstrated that over 80% of the respondents were aware of the most likely transmission routes, namely person to person spread via airborne droplets. This is in line with the information provided by the MoH website (Ministry of Health of New Zealand 2022). Relatively few respondents believed the incorrect statements that transmission was only from animals, or that the disease was not transmissible.

Ethnicity categories were re-grouped into three new groups - Chinese, Indians, and ‘other Asians including Filipinos’ to satisfy *Chi*-square test requirements. There was a significant difference between respondents’ perception of transmission of COVID-19 and ethnicity (χ^2^ = 154.8; df = 12; *p*<0.001). Table 4 shows that Chinese respondents were significantly more likely to believe transmission could occur through all transmission pathways, compared with the other ethnic groups, with the exception of transmission from animals to human only. Interestingly, as for the question of ‘COVID-19 is transmissible via droplets through coughing, sneezing or intimate contact’, significantly more ‘other Asians including Filipinos’ believed it than Indians.

**Table 4:** Significant differences of respondents’ answers regarding their perceptions of transmission of COVID-19

Which is the following do you think are correct about the transmission of COVID-19?	Ethnicity		
	Chinese	Indians	Other Asians including Filipinos
	Column N %	Column N %	Column N %
COVID-19 is transmissible from person to person	95.1%	79.7%	79.4%
COVID-19 is transmitted by animals to humans only	15.8%	16.2%	8.2%
COVID-19 is transmissible via droplets through coughing, sneezing or intimate contact	99.6%	79.2%	90.7%
COVID-19 is transmissible via the faecal-oral route	72.7%	43.0%	29.4%
COVID-19 is transmissible via food	56.1%	27.9%	29.9%
COVID-19 is transmissible via contaminated objects	82.3%	64.7%	57.0%

#### The perceived incubation period of COVID-19

**Fig. 5:**
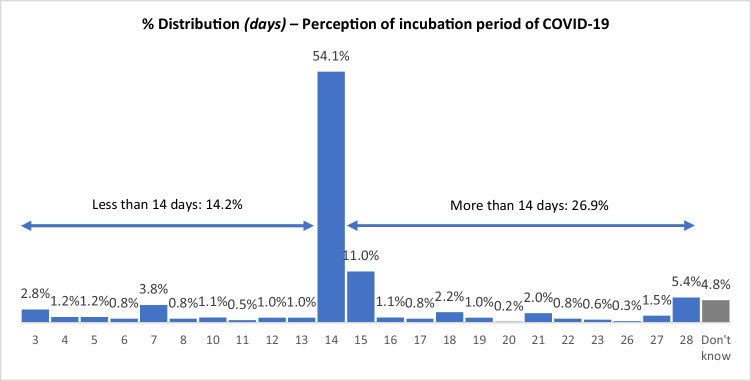
Distribution of the perceived incubation period of COVID-19

The current MoH information shows that the length of infectious period is approximately two days (Ministry of Health of New Zealand 2022); however, in 2020 when the survey was conducted, the median incubation periods of COVID-19 used for modelling in New Zealand was 5.1 days, and 97.5% of those who develop symptoms were within 11.5 days (Lauer et al. 2020). The survey found that the mean reported incubation period was 14.6 days (SD = 5.1days) (Fig. 5).

A Kruskal-Wallis rank sum test was performed to analyse the differences among ethnic groups. The result (χ^2^ = 31.607, df = 3, *p* < 0.001) indicated that there are statistically significant differences between perceived incubation period of COVID-19 and ethnicity (Table 5). Pairwise comparisons using a Wilcoxon rank sum test with continuity correction indicated that the reported incubation period of Chinese were significantly different from other ethnicities (Chinese vs. India, Filipinos or others, all p < 0.001). By comparison, Chinese perception of the incubation period of COVID-19 (average of 16.4 days) was significantly longer than other three ethnic groups (Indians - 14 days, Filipinos -12.7 days, and ‘other Asians’ - 13.2 days). There were no significant differences between gender and age group in terms of perceived incubation period.

**Table 5:** Significant differences of respondents’ answers regarding their perceptions of incubation period of COVID-19

	Ethnicity			
	Chinese	Indians	Filipinos	Other Asians
	Mean	Mean	Mean	Mean
What is the incubation period of COVID-19 (i.e., the time from viral infection to developing symptoms of illness)?	16.4 days	14 days	12.7 days	13.2 days

#### Perceived potential development of immunity to COVID-19

**Fig. 6:**
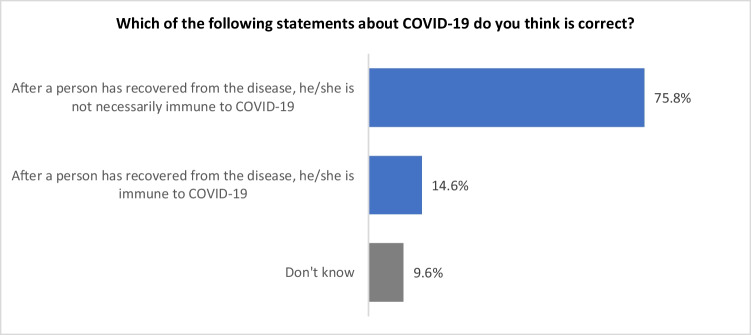
Distribution of perceived potential development of immunity to COVID-19

According to the MoH of New Zealand (2022), once a person is infected with COVID-19, their body will usually produce antibodies from a particular class of immune cell that ‘remember’ the virus. MoH assumed these antibodies give the person immunity from the virus, but it is not currently clear how long immunity lasts. A higher proportion (76%) of the Asian community believed that recovery from COVID-19 does not necessarily result in immunity, 14.6% of them believed ‘he/she is immune to COVID-19’, and 9.6% ‘do not know’ what would be the potential sequelae of COVID-19 (Fig. 6).

There are statistically significant differences between perceived potential immunity to COVID-19 and ethnicity (χ^2^ = 9.0; df = 2; *p* = 0.011). Table 6 shows that significantly more Indians (24.0%) than Chinese (10.4%) thought ‘after a person has recovered from the disease, he/she is immune to COVID-19’. In addition, significantly more Chinese (89.6%) than Indians (76%) thought ‘after a person has recovered from the disease, he/she is not necessarily immune to COVID-19’.

**Table 6:** Significant differences of respondents’ answers regarding their perceptions of potential sequelae of COVID-19

Which of the following statement(s) about COVID-19 do you think is correct?	Ethnicity		
	Chinese	Indians	Other Asians including Filipinos
	Column N %	Column N %	Column N %
After a person has recovered from the disease, he/she is not necessarily immune to COVID-19	89.6%	76.0%	85.7%
After a person has recovered from the disease, he/she is immune to COVID-19	10.4%	24.0%	14.3%

### Risk perceptions

#### Health impacts of COVID-19 on human

**Fig. 7:**
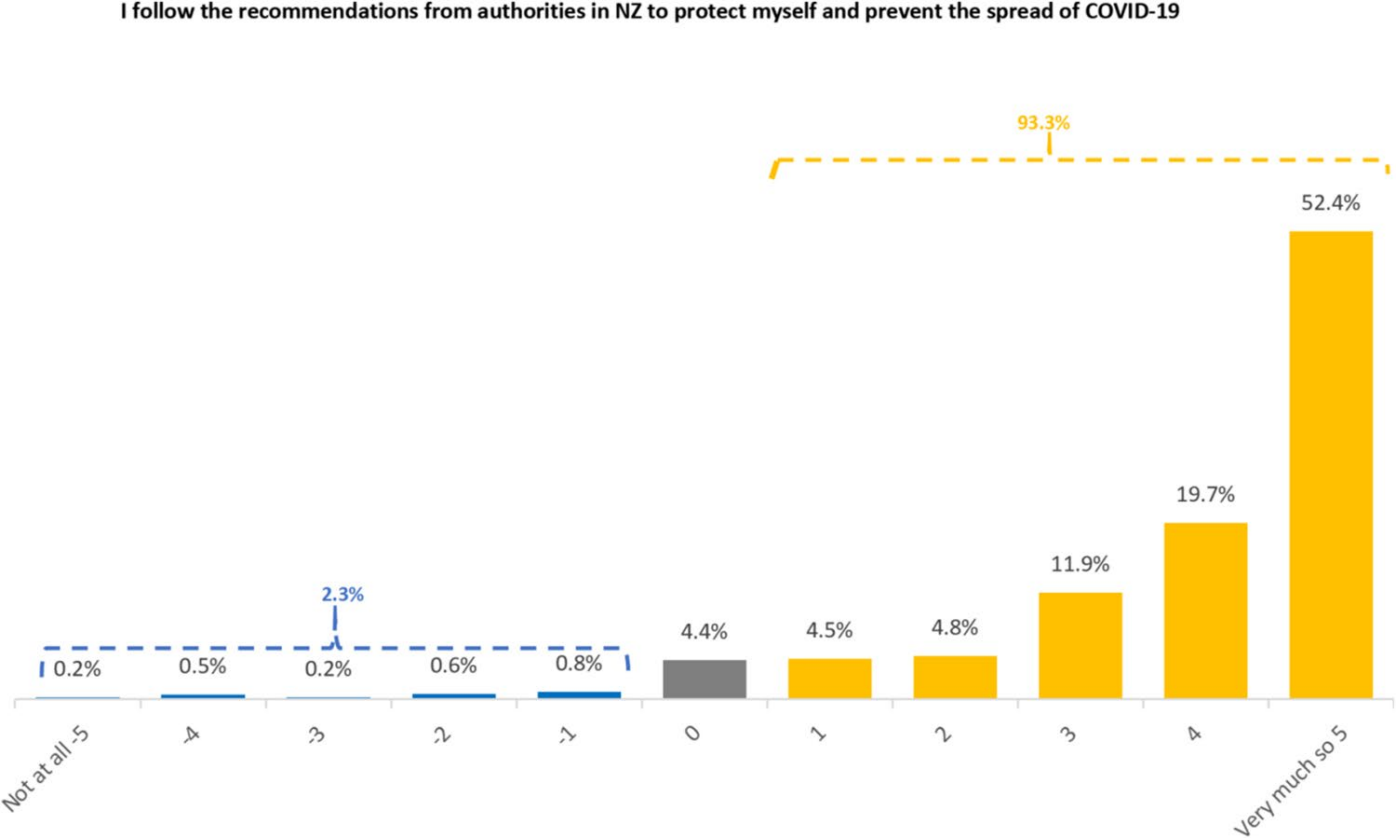
Distribution of perceived health impacts of COVID-19

Asian participants’ perceived health impacts of COVID-19 on human were measured on a 10-point Likert scale ranging from -5 to +5 (-5 refers to ‘it is a deadly disease’, ‘not at all susceptible’, and ‘not at all severe’ respectively in each question; +5 refers to ‘it is just like a common cold’, ‘very susceptible’, and ‘very severe’). Figure 7 shows that a large proportion of the Asian respondents perceived COVID-19 to be a deadly disease (82%). Within this, 29.3% perceive COVID-19 to be extremely deadly whilst 5.2% perceive it to be ‘not as deadly.’ Only 12.6% of the respondents perceived COVID-19 as low risk, and 5.9% held a neutral position. The overall mean rating is -2.63 (SD=2.56), hence an overwhelmingly negative (lean towards ‘deadly disease’) perception of the dangerousness of COVID-19.

The Kruskal-Wallis rank sum test indicated that there was a non-significant difference between ethnic groups (χ^2^ = 6.478, df = 3, *p* = 0.091), but a significant difference between genders (χ^2^ = 5.651, df = 1, *p* = 0.017). Significant differences have also been observed among different age groups (χ^2^ = 6.425, df = 2, *p* = 0.04), especially between young (18-29) and old (over 50) people (*p* = 0.043).

#### Susceptibility to infection with COVID-19

**Fig. 8:**
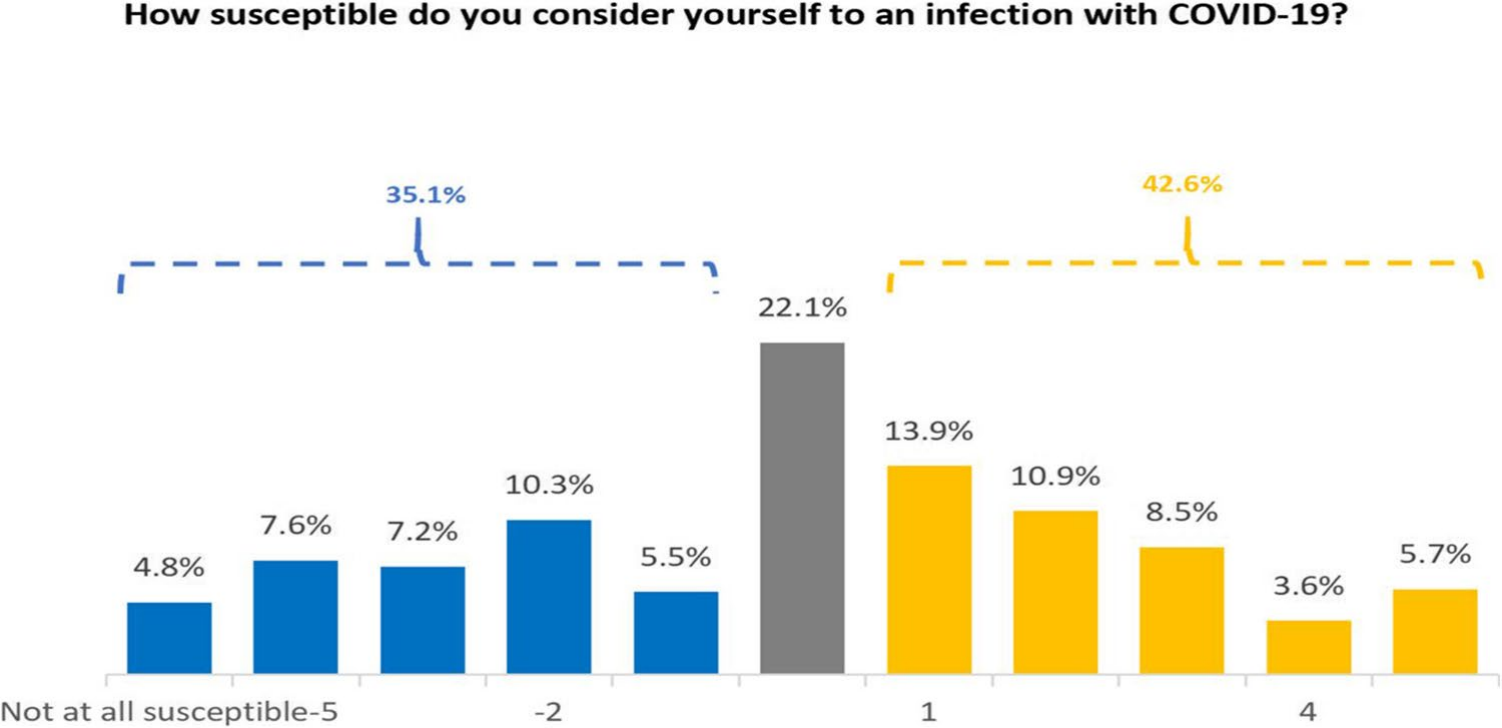
Distribution of perceived susceptibility of COVID-19

Perceptions of personal susceptibility to infection were widely spread from not at all susceptible to very susceptible (Fig. 8). The proportion of the respondents who thought they were susceptible to infection with COVID-19 (43%) was marginally higher than those who thought they were not susceptible (35%). Just over 20% of the respondents held a neutral position and the mean rating was close to zero (0.02, SD=2.6).

The Kruskal-Wallis rank sum test indicated that there was a non-significant difference between ethnic groups (χ^2^ = 7.07, df = 3, *p* = 0.0697) and between genders (χ^2^ = 3.11, df = 1, *p* = 0.0777). However, there was a significant difference between age groups (χ^2^ = 7.67, df = 2, *p* = 0.022). Young people (18-29) apparently perceived less susceptibility than other age groups (30-49 and 50+ groups, *p* = 0.030 and 0.043 respectively).

### Self-protection practices

#### Compliance behaviour

**Fig. 9:**
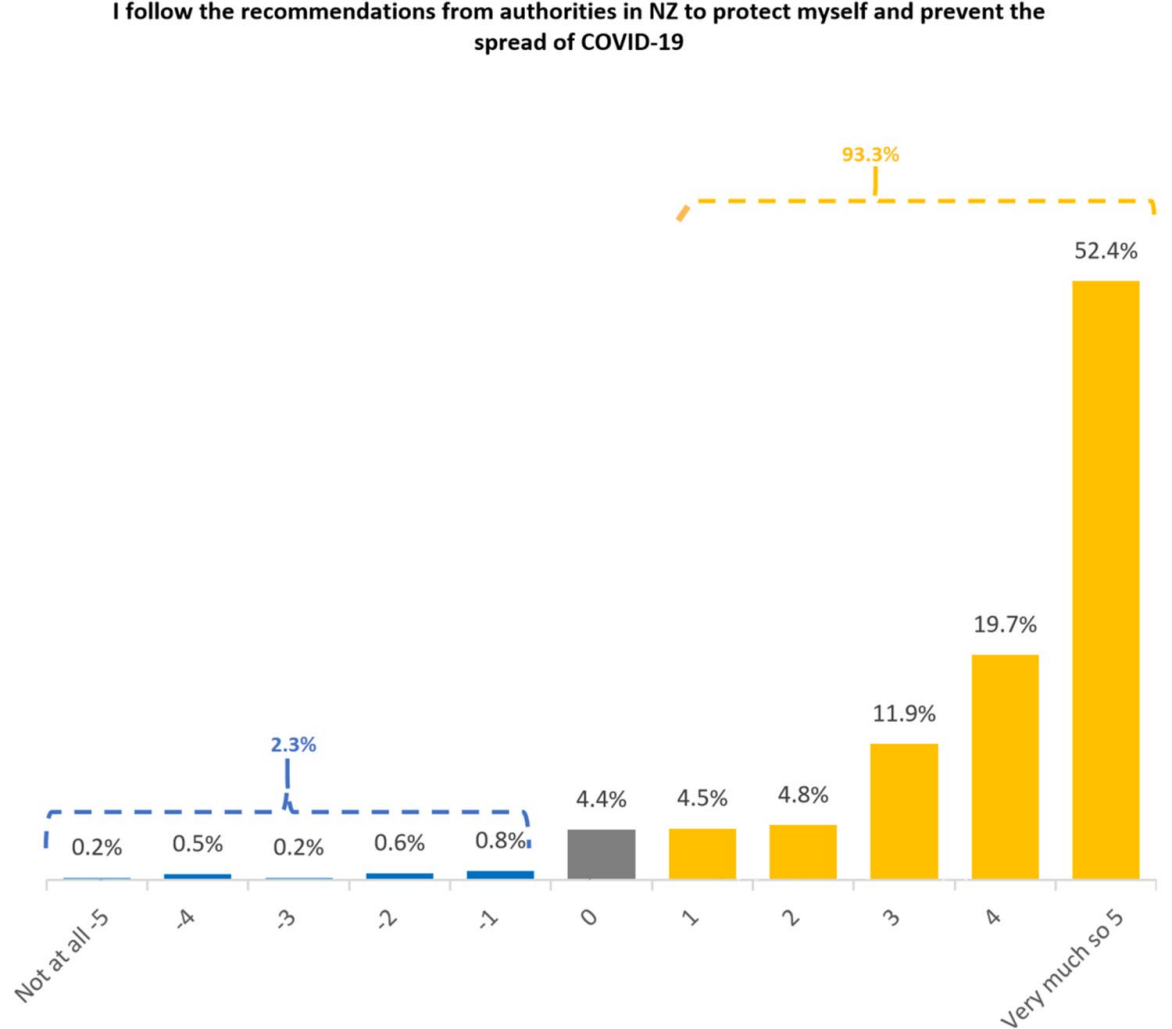
Distribution of compliance behaviour amongst the Asian Community

The vast majority of respondents (93.3%) reported following recommendations to protect themselves and prevent spread to others from relevant public health authorities in New Zealand. In addition, 52.4% self-reported that they followed these recommendations very closely; and overall, only 2.3% indicated some resistance to the government recommendations, while 4.4% of the respondents held a neutral position. The overall mean rating is 3.85 (SD=1.7), indicating a generally positive cooperation with recommendations from authorities (Fig. 9).

The Kruskal-Wallis rank sum test indicated that there was no significant differences among ethnic groups (χ^2^ = 5.136, df = 3, *p* = 0.162) or between genders (χ^2^ = 2.043, df = 1, *p* = 0.153). Similar to the above, there was a significant difference among different age groups (χ^2^ = 17.55, df = 2, *p* = 0.00015). Again, young people (18-29) were less likely to show compliance compared with the other age groups (30-49 and 50+ groups, *p* = 0.021 and 0.0001 respectively).

#### Self-protective practice

**Fig. 10:**
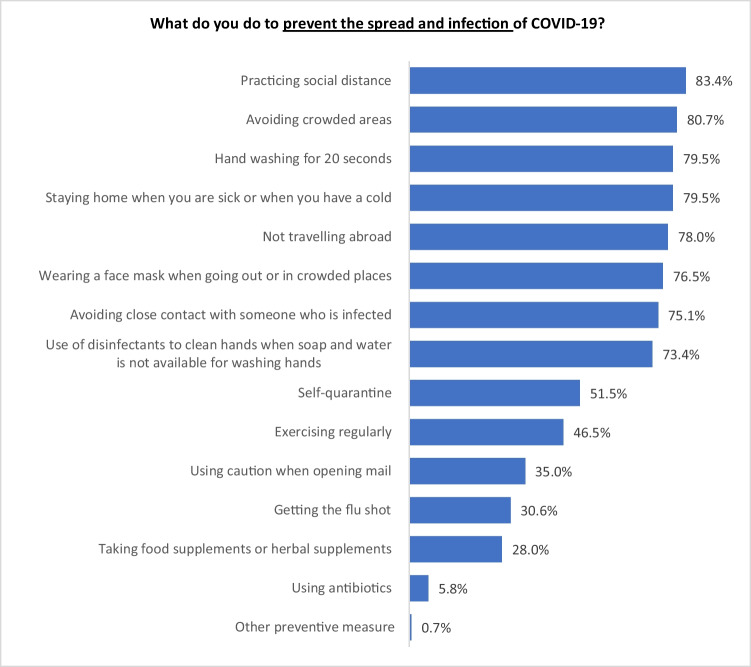
Distribution of self-protective practice amongst the Asian Community

Though 2.3% of the respondents reported that they were not fully following all the recommendations from relevant public health authorities in New Zealand, 100% of them reported that they adopted some form of self-protective practice to prevent the spread of and infection of COVID-19. The top five actions reported are: 1) social distancing (83.4%); 2) avoiding crowded places (80.7%); 3) handwashing for 20 seconds (79.5%); 4) staying home when sick (79.5%); and 5) not travelling abroad (78%). Other actions that align with recommendations from the health authorities were also reported to be practised by more than 70% of the respondents, such as wearing a face mask (Fig. 10).

Significant differences in reported self-protective practice were observed between ethnic (χ^2^ = 265.7; df = 42; *p*<0.001) and gender groups (χ^2^ = 42.8; df = 14; *p*<0.001) (Table 7). Table 7 shows that significantly more Chinese than Indians and ‘other Asian’ sub-groups reported that they adopted major preventive behaviours recommended by the authorities, including ‘use of disinfectants to clean hands when soap and water is not available for washing hands’, ‘staying home when you are sick or when you have a cold’, ‘avoiding crowded areas’, ‘avoiding close contact with someone who is infected’, ‘using caution when opening mail’, and ‘practicing social distance’. Strikingly, the proportion of choosing ‘wearing a mask when going out or in crowded places’ by Chinese is significantly higher than other three Asian sub-groups. There was also significantly more Chinese than Indians who chose ‘not travelling abroad’. Filipino occasionally demonstrated some significant differences in their answers with regard to some self-protection practices which were not compatible with the recommendations by relevant New Zealand authorities. For example, significantly more Filipinos than other three Asian sub-groups chose ‘taking food supplements or herbal supplements’, and ‘exercising regularly’. Apart from ethnicity, gender was also significantly associated with responses to some questions. For instance, more women than men chose ‘use of disinfectants to clean hands when soap and water is not available for washing hands’, ‘staying home when you are sick or when you have a cold’, ‘not travelling abroad’, ‘taking food supplements or herbal supplements’, and ‘avoiding close contact with someone who is infected’.

**Table 7:** Significant differences of respondents’ answers regarding their self-protection practice to prevent the spread and infection of COVID-19

What do you do to prevent the spread and infection of COVID-19?	Ethnicity				Gender	
	Chinese	Indians	Filipinos	Other Asians	Male	Female
	Column N %	Column N %	Column N %	Column N %	Column N %	Column N %
Hand washing for 20 seconds	81.7%	76.3%	91.0%	75.4%	79.9%	79.4%
Use of disinfectants to clean hands when soap and water is not available for washing hands	84.9%	58.5%	86.5%	71.5%	68.2%	78.1%
Staying home when you are sick or when you have a cold	89.6%	69.7%	86.7%	75.1%	72.7%	86.8%
Avoiding crowded areas	90.8%	72.1%	84.1%	76.5%	81.1%	80.7%
Not travelling abroad	84.2%	69.5%	84.5%	78.2%	71.4%	84.2%
Taking food supplements or herbal supplements	27.9%	23.5%	53.7%	22.9%	23.2%	32.4%
Wearing a face mask when going out or in crowded places	94.3%	61.6%	77.4%	70.5%	74.5%	78.7%
Avoiding close contact with someone who is infected	87.8%	65.0%	79.5%	68.2%	69.9%	79.9%
Using caution when opening mail	53.8%	25.7%	46.4%	13.4%	35.7%	34.2%
Getting the flu shot	31.9%	29.9%	35.2%	27.5%	32.9%	28.8%
Exercising regularly	58.8%	33.6%	60.7%	40.0%	50.0%	43.1%
Practicing social distance	92.0%	78.3%	84.3%	77.2%	84.1%	82.7%
Self-quarantine	52.7%	50.2%	53.3%	50.6%	53.8%	49.3%

## Results from the correlation analysis

**Fig. 11:**
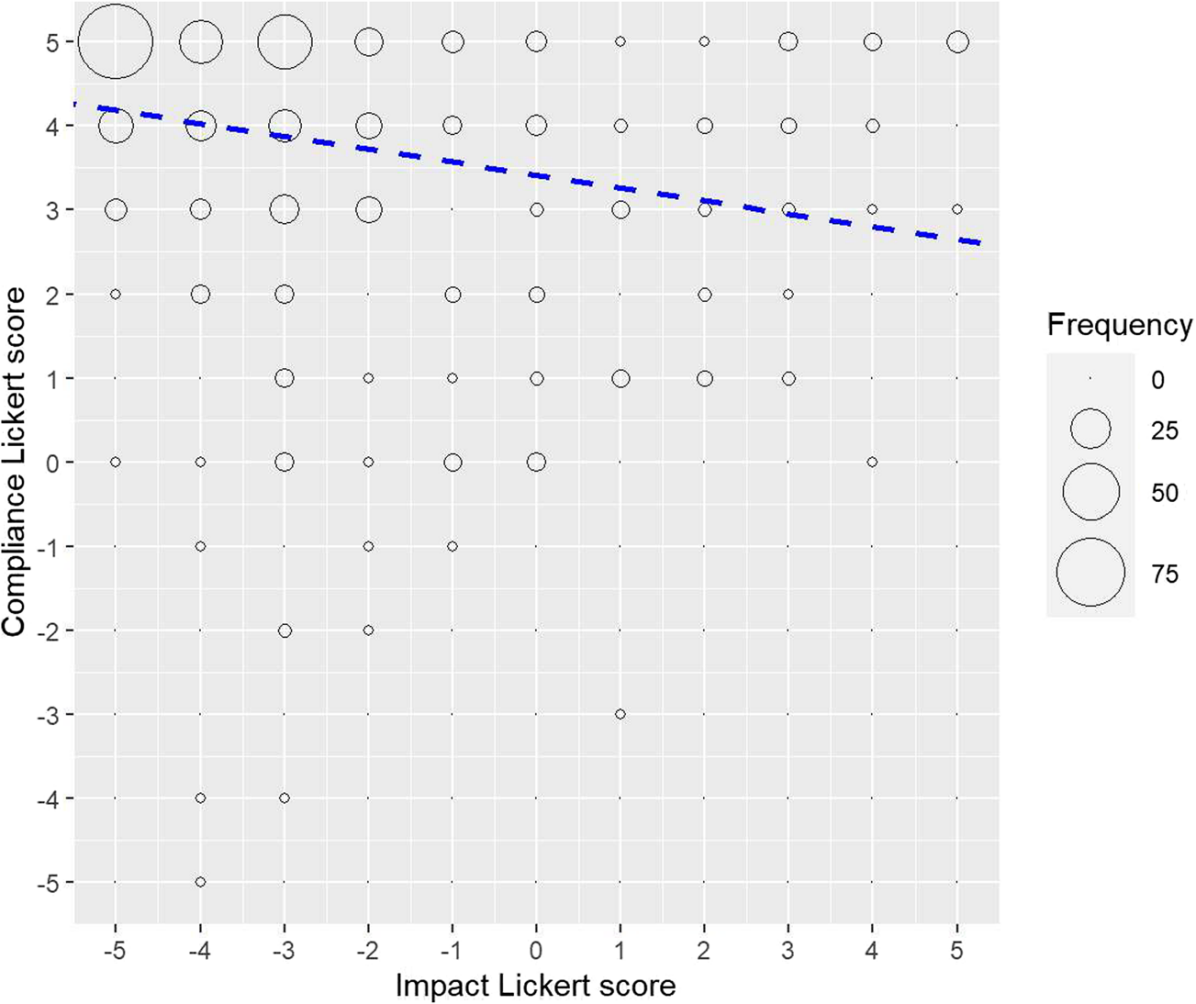
Correlation between the perceived health impact of COVID-19 on human and respondents’ overall reported compliance with preventive measures

The relationship between the perceived health impacts of COVID-19 on human (namely, ‘dangerousness’ of COVID-19 in the survey question) and respondents’ overall reported compliance with preventive measures (both on a Likert scale from -5 to 5) were compared using a linear regression analysis with an F-test (Fig. 11). There was a significant correlation between these two variables; i.e. a higher perception of ‘dangerousness’ (with lower value in Likert scale, i.e. -5) was associated with a higher reported compliance (higher value in Likert scale, i.e. +5) with preventive measures (correlation coefficient -0.22 (95% CI -0.31 to -0.13), F-statistic = 21.2 on 1 and 400 d.f , *p* < 0.0001).

## Conclusion and discussion

In general, the survey found high-risk perceptions and good knowledge of COVID-19 amongst Asians in New Zealand during the first year of the pandemic, and this is in line with research conducted in other countries (Jones et al. 2020; Nino et al. 2021; Niu et al. 2020). The majority of respondents showed a good knowledge of vulnerable populations, symptoms, asymptomatic transmission and potential sequelae of COVID-19. Secondly, the top five symptoms the respondents chose are fever, cough, shortness of breath, lost sense of smell, and sore throat, which is in agreement with the MoH’s advice. Thirdly, the Asian population had adequate knowledge of the transmission of COVID-19, with over 80% of respondents chose that COVID-19 can be transmitted are via ‘droplets’ and ‘person to person’. Lastly, as for the perceived potential sequelae of COVID-19, a high proportion of the respondents (76%) understand that recovery from COVID-19 does not necessarily result in immunity. These results largely aligned with the official information provided by the New Zealand Ministry of Health in 2020 when the survey was conducted.

In contrast, Asians’ understanding of the availability of a cure for, and the incubation period of COVID-19 was not consistent with the official information. The information provided on the MoH website was consistent with international data (Lauer et al. 2020) and suggests that ‘symptoms tend to arise around two to five days after a person has been infected but can take up to 14 days to show’ (Ministry of Health of New Zealand 2022). However, 54.1% of respondents believed that the incubation period was 14 days, and only14.2% respondents believed that the incubation period was less than 14 days. The discordance may have arisen due to a misinterpretation of the wording on the MoH website.

The survey found that most Asians’ perceived the health impacts of COVID-19 on human to be very high. However, the perceived high health impact of COVID-19 on human was not matched by a perception of high risk of contracting the disease.

The survey showed that Asians’ perceived COVID-19 to be a serious public health threat that required compliance with the health authorities’ recommendations to prevent transmission. A total of 93.3% of respondents reported that they followed recommendations, and 100% reported adopting some form of self-protective practice. Notably, compared to the national average percentage of wearing masks in 2020, the percentage of Asians who reported wearing masks in our survey was much higher; Gray et al. in 2020 showed that the national average of wearing masks during the first COVID-19 Alert Level 4 lockdown in 2020 was only 33.1% (Gray et al. 2020). The reason for the low average mask use in 2020 may be because the New Zealand government followed the WHO’s COVID-19 response guidelines which, at that time, suggested that masks were not necessary as a preventive measure. It was not until 30 August 2021 that the first nationwide face mask mandate on public transport was announced by New Zealand authorities. On the 19^th^ November 2021, face mask were mandated on public transport in Auckland, and nationwide for domestic flights. On the 25^th^ January 2022, as Omicron cases increased, a new mask rule was enforced which required medical grade masks to be worn at most indoor settings and close proximity businesses, events and gatherings (Moodie 2022). However, the survey was conducted before these recommendations and policies were introduced. Our survey indicates that Asians are more likely to wear a mask than the rest of the population, even before the government mandated mask wearing. While different cultural norms might contribute to the different attitude towards mask wearing, previous experience with a highly contagious respiratory disease may explain the high prevalence of mask wearing among the Asian populations. For example, during the 2012 SARS outbreak, wearing a mask was recommended by a number of Asian governments as a self-protection method (Ding et al. 2016; Drife 2003; Leung 2020; Lo et al. 2005).

There were some significant differences in responses to survey questions between different ethnic, age and gender groups. Ethnicity was associated varying responses to many questions, with the exception of perceived health impacts of COVID-19 on human, susceptibility to infection with COVID-19, and compliance behaviour. Among all sub-groups of the Asian respondents, Chinese risk perception of COVID-19 was highest, and in general their answers to COVID-19 knowledge questions were more consistent with the information published by relevant New Zealand authorities at the time the survey was conducted. Interestingly, Chinese perception of the incubation period (16.4 days) is significantly longer than other Asian sub-groups. The tendency to report higher uptake of preventive measures in Chinese may be due to their greater perception of risk. Research conducted by Bowman et al. in Hong Kong and UK (2021), by Wegemer and Keyserlingk in the US (2022), and by Gray et al. in New Zealand (2020) shows the similar behavioural trend. Apart from Chinese, Indian and Filipino sub-groups occasionally demonstrated some significant differences in their answers, including their answers to questions about potential sequelae of COVID-19 and self-protection practices. Those answering patterns are not in line with information provided by relevant New Zealand authorities.

The findings from this survey underline the diversity and important differences between Asian ethnic groups. ‘Asian’ sub-groups appeared to respond to the pandemic very differently. Therefore, it is problematic that public health statistics often group these sub-groups together as one single ethnic group without recognising the inherent heterogeneity and diversity. Such an arbitrary ethnic divide in public statistics is prevalent, not only in New Zealand (Steyn et al. 2021), but also in many other English-speaking countries, such as the UK and the US (Labgold et al. 2020; Martin et al. 2020; Pagel and Yates 2021). In policy making, it might result in a poor evidence-base for public health decision making and interventions. As a result, this may exacerbate health inequities and result in poorer outcomes for some ethnic groups.

The survey also provided evidence of differences in knowledge and beliefs between genders and age group. For example, Asian females tended to rank the health impacts of COVID-19 on human higher than Asian males, and significantly more Asian females than males adopted the recommended self-protection practices. These findings are consistent with international research which shows that women generally evaluate their COVID-specific risk more highly than men (Dryhurst et al. 2020; Lewis and Duch 2021; Petzold et al. 2020). In addition, compared with the Asian males, the answers for questions about the COVID-19 symptoms from Asian females were significantly more in agreement with the official information provided by relevant New Zealand authorities.

As for the age variable, significantly more respondents from the age group of over 50 years old perceived significantly higher dangerousness of COVID-19 compared with the age group of 18-29 years old. Significantly more young people aged between 18 and 29 years old perceived less susceptibility to infection with COVID-19 and reported that they ‘follow the recommendations from the authorities in New Zealand to prevent the spread of COVID-19’ compared with older age groups of 30-49 and 50+ years old. This finding about the age difference is in agreement with many international research which shows that younger adults perceived less susceptibility to infection with COVID-19 and were less likely to implement preventative measures (Atchison et al. 2021; Machida et al. 2020; Wolfe et al. 2021). Finally, the correlation analysis shows the higher perceived dangerousness of COVID-19, the closer compliance to self-protection practices among New Zealand Asian population.

The research had several limitations which need to be acknowledged. First of all, the sample size is modest, which reduces the precision of our estimated responses and the power to detect differences between demographic groups. Secondly, although a common practice, the survey questions are self-reporting in nature, which is prone to a range of information biases, such as recall, social undesirability and end aversion bias which commonly exist in responses to Likert scale questions. Lastly, the COVID-19 situation was and still is rapidly evolving, therefore the responses reflect the situation during the early stages of the pandemic and not the current situation. Asian perceptions of COVID-19 may have changed as the pandemic progressed and different prevention and control measures were introduced. A follow-up survey will be more informative to reflect the current situation and changes.

## Appendix

**Table 8:** The sample composition breakdown

	(%) Sample Distribution	(n) Sample size
Gender		
Male	49.0%	197
Female	50.5%	203
Other	0.5%	2
Country of Origin		
Mainland China	31.3%	126
Hong Kong	2.0%	8
Taiwan	1.3%	5
Vietnam	1.5%	6
Cambodia	1.3%	5
India	33.6%	135
The Philippines	10.3%	41
Korea	5.0%	20
Japan	2.5%	10
Sri Lanka	2.3%	9
Singapore	2.1%	8
Malaysia	4.7%	19
Thailand	0.9%	4
Other Asian countries	1.3%	5
Age Groups		
18-29 years	33.4%	134
30-49 years	35.1%	141
50-64 years	23.4%	94
65 years +	8.1%	33
Region		
Northland Region	1.6%	6
Auckland Region	65.1%	262
Waikato Region - Hamilton	3.8%	15
Waikato Region - other	0.2%	1
Bay of Plenty Region	1.6%	6
Hawke's Bay Region	0.7%	3
Taranaki Region	1.1%	4
Manawatu-Wanganui Region	2.2%	9
Wellington Region	12.8%	52
Tasman Region	0.3%	1
Marlborough Region	0.5%	2
Canterbury Region - Christchurch	7.0%	28
Canterbury Region - other	0.5%	2
Otago Region - Dunedin	0.8%	3
Otago Region - Queenstown	0.5%	2
Otago Region - other	0.3%	1
Southland Region	1.0%	4
Total	**100%**	**402**
